# Global burden of rheumatoid arthritis among adolescents and young adults aged 10–24 years: A trend analysis study from 1990 to 2019

**DOI:** 10.1371/journal.pone.0302140

**Published:** 2024-04-16

**Authors:** Ruibo Li, Xingyue Yuan, Yili Ou

**Affiliations:** 1 Department of Orthopaedics, Deyang Peoples’ Hospita, Deyang, Sichuan, China; 2 Deyang Clinical College, Chengdu University of Traditional Chinese Medicine, Deyang, Sichuan, China; 3 Department of Pathology, Deyang Peoples’ Hospita, Deyang, Sichuan, China; Atal Bihari Vajpayee Institute of Medical Sciences & Dr Ram Manohar Lohia Hospital, INDIA

## Abstract

**Background:**

In recent decades, there has been a global increase in the burden of rheumatoid arthritis (RA) among adolescents and young adults (AYAs), making it a significant public health issue. However, our understanding of the disease burden, harm, and influencing factors of RA in this population remains insufficient. This study aimed to assess the trends in RA burden among AYAs aged 10–24 years from 1990 to 2019 at the global, regional, and national levels.

**Methods:**

Incidence, prevalence, and disability-adjusted life years (DALYs) rate per 100,000 population, as well as average annual percentage changes (AAPCs), of RA among individuals aged 10–24 years were reported globally, regionally, and nationally based on the Global Burden of Diseases, Injuries, and Risk Factors Study 2019 (GBD 2019). These global trends were further analyzed by age, sex, and Sociodemographic index (SDI). Joinpoint regression analysis was used to determine the year in which the most significant changes in global trends occurred.

**Results:**

Globally, the incidence of RA among AYAs increased from 4.98 per 100,000 population in 1990 to 5.41 per 100,000 population in 2019, with an AAPCs of 0.29 (95%CI: 0.26, 0.32, p < 0.001). The most significant increase occurred in 2000, while the most significant decrease occurred in 2014. The prevalence increased from 34.11 per 100,000 population in 1990 to 36.34 per 100,000 population in 2019, with an AAPCs of 0.22 (95%CI: 0.19, 0.24, p<0.001); The most significant increase was observed in 2000, and the most significant decrease occurred in 2014. DALYs rate with RA were 5.96 per 100,000 population in 1990 and 5.79 per 100,000 population in 2019 for AYAs, with an average decrease of 0.1 years per year (AAPCs = -0.1, 95%CI: -0.2, -0.01, p = 0.04). In terms of gender, the incidence, prevalence, and DALYs rate were higher for females compared to males during the same period. Regarding age, the incidence, prevalence, and DALYs rate increased with increasing age. Based on the SDI quintile, the incidence, prevalence, and DALYs rate of RA were highest in countries with high SDI and lowest in countries with low SDI from 1990 to 2019. However, the relationship between incidence and SDI is non-linear. In terms of regions, Tropical Latin America exhibited the highest incidence, prevalence, and DALYs rate, while Andean Latin America experienced the most rapid increase in incidence and prevalence. Southern Latin America saw the fastest growth in DALYs rate, whereas Southern Sub-Saharan Africa witnessed the most significant decline.

**Conclusion:**

In conclusion, the study revealed an overall increase in the incidence and prevalence of RA among adolescents and young adults (AYAs) over the past three decades, while DALYs rate remained relatively stable. Furthermore, the incidence, prevalence, and DALYs rate of RA were found to increase with age. Fortunately, recent proactive preventive measures and treatment methods have shown promising results. Moving forward, it is crucial to prioritize the female population and AYAs patients in order to further alleviate the global burden of RA.

## Introduction

Rheumatoid arthritis (RA) is a chronic autoimmune disease characterized by inflammation and joint damage, leading to pain, disability, and decline quality of life. Although RA is commonly associated with older adults, recent evidence suggests that it also affects a substantial number of adolescents and young adults (AYAs) [1, 2]. Over the past few decades, the burden of RA among AYAs has increased globally, making it a significant public health issue. The National Health Interview Survey (NHIS) in the United States estimates that the annual prevalence of arthritis among individuals under the age of 25 is 1.3%, indicating that there are nearly 400,000 children, adolescents, and young adults affected by arthritis in the United States [3].

Studies have shown that patients with RA require ongoing medical care and medication, which not only increases medical expenses, but also leads to a loss of work ability and quality of life. In addition, young people with RA may develop complications such as cardiovascular disease, mental health problems, often resulting in missing school. In severe cases, these complications can result in long-term physical disabilities that persist into adulthood [4]. Despite advances in treatment with disease-modifying anti-rheumatic drugs and biologics, most AYAs with RA continue to experience active disease in adulthood, further increasing the cost of treatment and management [5]. Socioeconomic inequality, characterized by disparities in income, education level, and access to healthcare services, has been shown to influence health outcomes for various diseases [6–8]. Multiple studies support a positive association between low-income, low socioeconomic status, or low educational status and non-communicable diseases [7]. However, Safiri S et al. [9] found that social development index was non-linear correlated with age-standardized DALYs in rheumatoid arthritis. Until now, the specific impact of socioeconomic inequality on the burden of RA among AYAs remains understudied. Therefore, it is crucial to pay attention to the prevention, diagnosis, and treatment of RA in this vulnerable population.

In this study, we comprehensively assessed the global burden and trends of RA among individuals aged 10–24 years in 204 countries and territories, taking into account factors such as geographical region and socio-demographic development. The findings of this study contribute to the existing literature by providing valuable information for healthcare policies and resource allocation, as well as guiding the development of prevention and intervention strategies to effectively reduce the disease burden of RA in AYAs.

## Materials and methods

### Study population and data collection

The Burden of Diseases, Injuries, and Risk Factors Study 2019 (GBD 2019) was published by the Institute for Health Metrics and Evaluation (IHME), based in the United States [7]. All data is open source and available to the public. It is the most comprehensive and detailed study of global diseases, injuries, and risk factors. The GBD 2019 covers 204 countries and territories and estimates the burden of 369 diseases and injuries from 1990 to 2019, including RA. The introduction and estimation methods of GBD 2019 are described in detail in the previous systematic analysis study for the Global Burden of Disease [10]. All data used in this study were obtained from publicly available databases; further ethical approval was not required.

The United Nations defines adolescence as the period between the ages of 10 and 19 years [11]. However, there is no precise definition of young adult at present, and it is often defined as the age range of 15 to 24 years. In the GBD 2019 database, age groups include 10–14 years, 15–19 years, and 10–24 years. Taking into account the GBD 2019 database age group and the general concept, we define the age group of AYAs as 10–24 years old. Furthermore, a definition of 10–24 years corresponds more closely to adolescent growth and popular understandings of this life phase and would facilitate extended investments across a broader range of settings [12]. Following the definition of the GBD project, we grouped data on RA based on the age group of 10–24 years and 21 geographically close and epidemiologically similar country regions to provide a more detailed description of the disease burden of RA among patients in this age range. The 21 geographic regions include: Andean Latin America, Australasia Caribbean, Central Asia, Central Europe Central Latin America, Central Sub-Saharan Africa, East Asia, Eastern Europe, Eastern Sub-Saharan Africa, High income Asia Pacific, High income North America, North Africa and Middle East, Oceania, South Asia, Southeast Asia, High income Asia Pacific, High income North America, North Africa and Middle East, Oceania, South Asia, Southeast Asia, Southern Latin America, Southern Sub-Saharan Africa, Tropical Latin America, Western Europe, and Western Sub-Saharan Africa.

The study focused primarily on the trends of three indicators: incidence, prevalence, and disability-adjusted life years (DALYs). DALYs, a measure of disease burden, combines years of life lost due to premature mortality and years lived with disability caused by the disease. The DALYs of RA in AYAs group were calculated using the relevant data provided by GBD 2019, and the trend was further analyzed.

The GBD 2019 also calculated the sociodemographic index (SDI) for each country, which is a composite measure of social and economic conditions that influence health outcomes in each region. SDI measures the overall economic development of a society based on indicators such as education level, per capita income, and fertility rate. The SDI ranges from 0 to 1, with higher values representing higher levels of socioeconomic development. SDI is divided into five quintiles: low, low-middle, middle, high-middle, and high [10]. The relationship between the SDI and the incidence in different countries and regions was analyzed in this study.

### Statistical analysis

The data analysis method used in this paper refers to the method of Zhang Jing et al [13]. Incidence, prevalence, and DALYs rate for RA in AYAs were obtained from the cross-sectional data of GBD 2019, which provided estimates with 95% uncertainty intervals (UI), representing the 25th and 975th ordered estimates derived from 1,000 draws from the posterior distribution. Age-specific ratios and their annual mean percentage changes (AAPCs) were calculated by linear regression using the logarithm of the ratio on a logarithmic scale as the dependent variable and the year as the independent variable. AAPCs is a summary measure of the trend over a pre-specified fixed interval, calculated as the weighted average of the annual percent changes (APCs), allowing us to describe the average APCs over the study period with a single number. The value of AAPCs represents the annual percentage change (increase, decrease, or no change). For example, if the AAPCs is 0.1, it means there is a 0.1% increase in the annual growth rate. The trend in the incidence was reflected in the AAPCs value and its 95% confidence interval. The AAPCs for incidence, prevalence, and DALYs rate from 1990 to 2019 were calculated. Additionally, the years with the most substantial changes in trends for the aforementioned indicators were determined. Joinpoint regression analysis was employed to identify trends in the data over time, with the simplest model being fitted by connecting several different line segments on a logarithmic scale. These segments, known as joinpoints, were tested using a Monte Carlo permutation method. The final model was selected using the weighted Bayesian information criteria method and the suggestions provided by Joinpoint software. Global trends were analyzed, and stratified analyses of these trends were conducted by age group, gender, and SDI. The methodology used in AAPCs calculations was the same as described above. All statistical analyses were performed using RStudio software (version 4.3.1) and the Joinpoint Regression Program (version 5.0.2).

## Results

### Incidence

Overall, the global incidence of RA in AYAs slightly increased from 4.98 per 100 000 population in 1990 to 5.41 per 100 000 population in 2019, with an AAPCs of 0.29 (95%CI: 0.26, 0.32), indicating a significant positive trend (p < 0.001). When comparing genders, both males and females experienced an increase in incidence. However, the rate of increase was higher for males (AAPCs = 0.40, 95%CI: 0.36, 0.43) than for females (AAPCs = 0.25, 95%CI: 0.22, 0.28). In terms of age groups, the incidence generally increased from 1990 to 2019, although the rate of increase varied across different age ranges. The highest rate of increase was observed in the 20–24 years age group (AAPCs = 0.32, 95%CI: 0.29, 0.36), followed by 15–19 years (AAPCs = 0.27, 95%CI: 0.24, 0.29) and 10–14 years (AAPCs = 0.16, 95%CI: 0.13, 0.18).

The incidence varies with different SDI. Overall, the higher the SDI, the higher the incidence. In high SDI areas, the incidence was 6.05 per 100 000 population (95%UI: 4.80, 7.74) in 1990 and 7.02 per 100 000 population (95%UI: 5.59, 8.95) in 2019, while in low SDI areas, the incidence was 3.28 per 100 000 population (95%UI: 2.39, 4.47) in 1990 and 3.49 per 100 000 population (95%UI: 2.52, 4.77) in 2019. At the same time, the average increase in incidence in high SDI areas was 0.52% (95%CI: 0.50, 0.54, p < 0.001), while the average increase in incidence in low SDI areas was 0.22% (95%CI: 0.20, 0.24, p < 0.001).

At the regional level, the incidence has increased significantly in all regions except East Asia and Southern Sub-Saharan Africa. In East Asia, there was a slight but not statistically significant increase in incidence (AAPCs = 0.14, p = 0.24). In Southern Sub-Saharan Africa, there was a slight downward trend in incidence (AAPCs = -0.14), but no statistical difference (p = 0.07) (Table 1).

**Table 1:** The incidence and AAPCs of rheumatoid arthritis among adolescents and young adults from 1990 to 2019.

	Incidence per 100000 population (95% UI) in 1990	Incidence per 100000 population (95% UI) in 2019	AAPCs (95% CI), 1990 to 2019	P value *
**Global**	4.98 (3.76, 6.51)	5.41 (4.09, 7.09)	0.29 (0.26, 0.32)	< 0.001
**Sex**				
Male	2.90 (2.17, 3.87)	3.26 (2.45, 4.32)	0.40 (0.36, 0.43)	< 0.001
Female	7.12 (5.43, 9.24)	7.66 (5.82, 10.00)	0.25 (0.22, 0.28)	< 0.001
**Age group**				
10–14 years	3.15 (2.35, 4.06)	3.30 (2.46, 4.28)	0.16 (0.13, 0.18)	< 0.001
15–19 years	5.17 (3.64, 7.27)	5.62 (3.96, 7.93)	0.27 (0.24, 0.29)	< 0.001
20–24 years	6.76 (5.12, 8.75)	7.45 (5.64, 9.71)	0.32 (0.29, 0.36)	< 0.001
**Sociodemographic index**				
High	6.05 (4.80, 7.74)	7.02 (5.59, 8.95)	0.52 (0.50, 0.54)	< 0.001
High-middle	5.39 (4.12, 7.04)	6.31 (4.83, 8.19)	0.54 (0.52, 0.56)	< 0.001
Middle	5.04 (3.77, 6.65)	5.67 (4.24, 7.47)	0.40 (0.33, 0.47)	< 0.001
Low-middle	4.75 (3.58, 6.24)	5.52 (4.18, 7.21)	0.51 (0.46, 0.56)	< 0.001
Low	3.28 (2.39, 4.47)	3.49 (2.52, 4.77)	0.22 (0.20, 0.24)	< 0.001
**Region**				
Andean Latin America	6.57 (4.44, 9.67)	9.09 (6.15, 13.29)	1.12 (1.10, 1.14)	< 0.001
Australasia	5.53 (3.73, 8.06)	6.68 (4.60, 9.68)	0.66 (0.63, 0.68)	< 0.001
Caribbean	7.23 (4.93, 10.48)	8.60 (5.89, 12.54)	0.59 (0.54, 0.65)	< 0.001
Central Asia	6.83 (4.71, 9.86)	8.49 (5.85, 12.19)	0.73 (0.61, 0.85)	< 0.001
Central Europe	4.81 (3.45, 6.65)	5.86 (4.23, 8.15)	0.70 (0.67, 0.72)	< 0.001
Central Latin America	8.23 (6.30, 10.75)	10.02 (7.61, 13.11)	0.67 (0.62, 0.73)	< 0.001
Central Sub-Saharan Africa	2.87 (1.83, 4.39)	3.05 (1.95, 4.71)	0.20 (0.12, 0.27)	< 0.001
East Asia	5.07 (3.90, 6.55)	5.31 (4.10, 6.80)	0.14 (-0.09, 0.36)	0.24
Eastern Europe	4.52 (3.44, 5.84)	5.09 (3.87, 6.59)	0.40 (0.28, 0.51)	< 0.001
Eastern Sub-Saharan Africa	2.58 (1.82, 3.70)	2.75 (1.91, 3.95)	0.23 (0.15, 0.31)	< 0.001
High-income Asia Pacific	4.97 (3.81, 6.34)	5.91 (4.56, 7.59)	0.58 (0.52, 0.64)	< 0.001
High-income North America	5.83 (5.24, 6.53)	6.91 (6.12, 7.91)	0.60 (0.55, 0.65)	< 0.001
North Africa and Middle East	3.66 (2.58, 5.17)	4.42 (3.06, 6.27)	0.66 (0.63 to 0.69)	< 0.001
Oceania	3.71 (2.49, 5.45)	3.89 (2.62, 5.85)	0.16 (0.15, 0.18)	< 0.001
South Asia	4.88 (3.70, 6.27)	5.97 (4.54, 7.62)	0.69 (0.68, 0.70)	< 0.001
Southeast Asia	3.07 (2.22, 4.24)	3.42 (2.49, 4.69)	0.37 (0.35, 0.38)	< 0.001
Southern Latin America	5.11 (3.39, 7.58)	6.46 (4.31, 9.68)	0.81 (0.79, 0.83)	< 0.001
Southern Sub-Saharan Africa	6.41 (4.99, 8.17)	6.22 (4.79, 8.03)	-0.14 (-0.29, 0.01)	0.07
Tropical Latin America	11.91 (9.47, 14.98)	14.49 (11.59, 18.00)	0.68 (0.66,o 0.69)	< 0.001
Western Europe	7.23 (5.25, 10.04)	7.96 (5.77, 10.95)	0.33 (0.31, 0.35)	< 0.001
Western Sub-Saharan Africa	1.32 (5.25, 1.95)	1.56 (1.06, 2.27)	0.55 (0.46, 0.65)	< 0.001

UI = uncertainty interval. AAPCs = average annual percentage changes. CI = confidence interval.

* The P-value was determined by joinpoint regression analysis.

**Fig 1:**
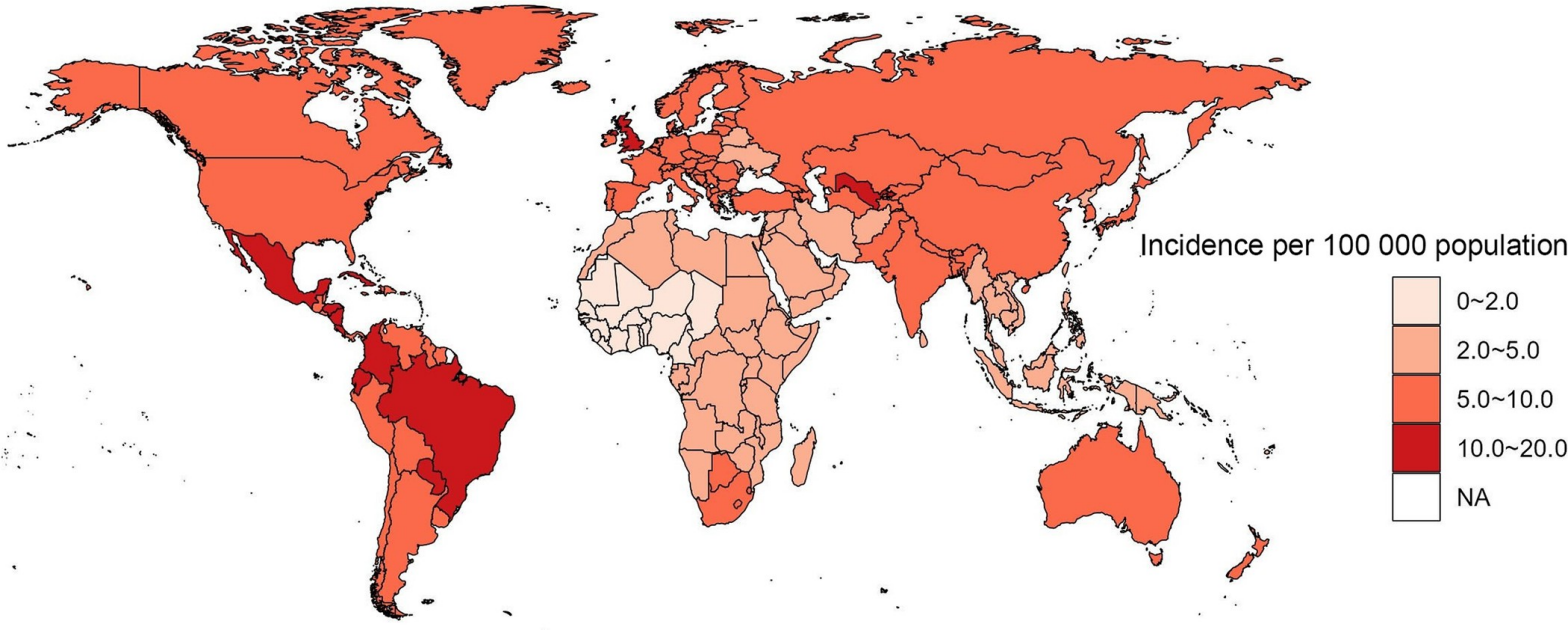
Global map of 2019 incidence of RA among AYAs. Map created in R using rmaps package (https://cran.r-project.org/web/packages/maps/index.html) and data from the 2019 incidence of rheumatoid arthritis among adolescents and young adults [https://vizhub.healthdata.org/gbd-results/]. RA = rheumatoid arthritis. AYAs = adolescents and young adults.

Fig 1 summarizes the global incidence of RA in AYAs at the country level in 2019. The countries with the lowest incidence of RA in AYAs in 2019 were Chad (1.37 per 100 000 population, 95% UI: 0.86, 2.17), while Brazil had the highest incidence at 14.64 per 100 000 population (95% UI: 11.73, 18.09).

**Fig 2:**
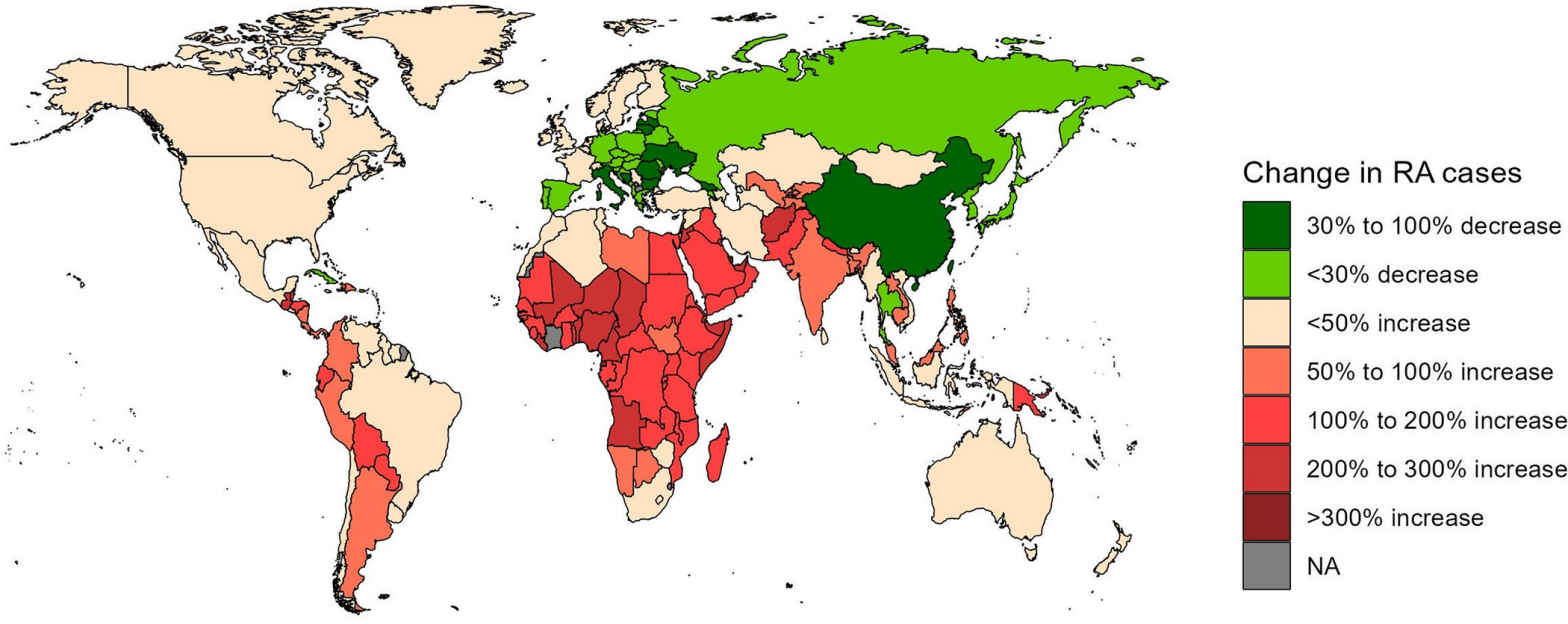
The relative change in incident cases of RA among AYAs between 1990 and 2019. Map created in R using rworldmaps package (https://cran.r-project.org/web/packages/rworldmap/index.html) and Natural Earth data (http://www.naturalearthdata.com/). RA = rheumatoid arthritis. AYAs = adolescents and young adults.

Fig 2 shows the rate of change in the number of new cases in different countries from 1990 to 2019. Qatar topped the list with a 523% increase, followed by Equatorial Guinea with a 413% increase. Forty-six countries saw a decline in the number of new cases of RA among AYAs from 1990 to 2019, with a 48% decline in Georgia and a 43% decline in Bulgaria.

**Fig 3:**
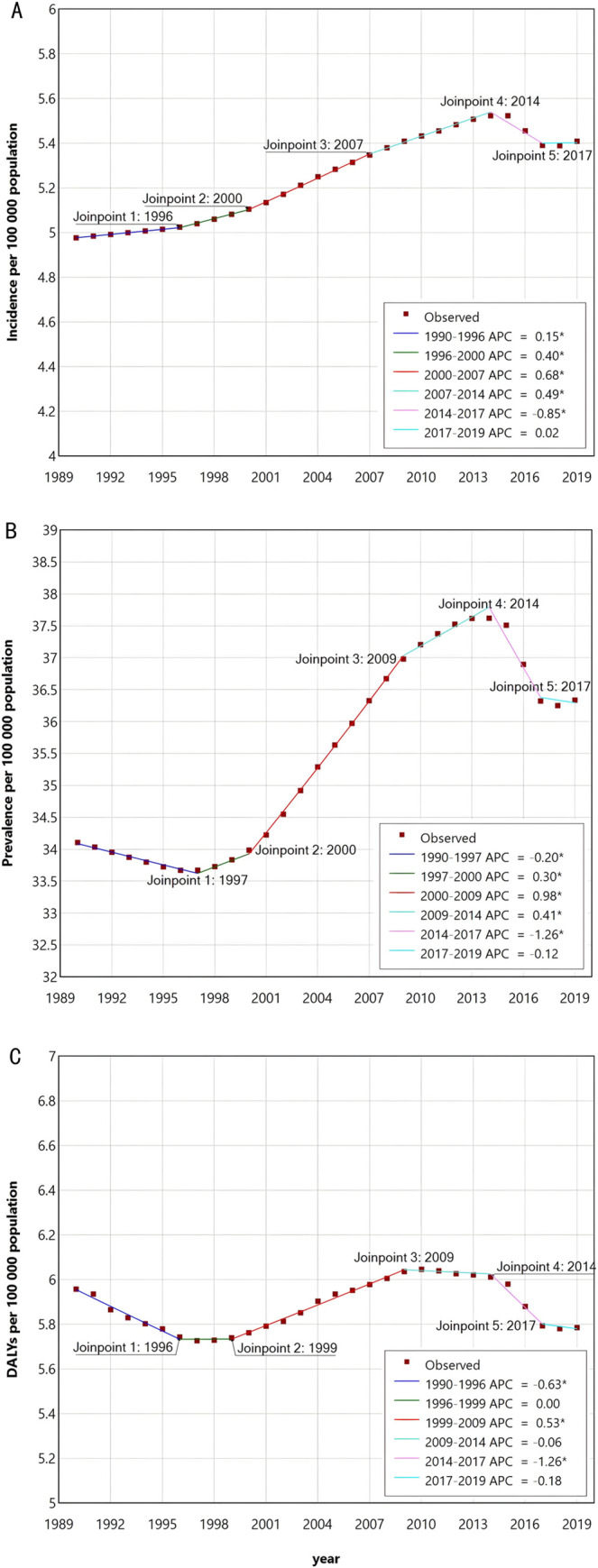
Joinpoint regression analysis of global RA incidence (A), prevalence (B), and DALYs rate (C) in AYAs aged 10–24 years from 1990 to 2019. * Indicates that the APC is significantly different from zero at the alpha = 0.05 level. RA = rheumatoid arthritis. DALYs = disability-adjusted life-years. AYAs = adolescents and young adults. APC = annual percentage change.

The joint point regression analysis found substantial changes in the incidence of RA in 1996, 2000, 2007, 2014 and 2017, with the largest change occurring in 2014 (Fig 3A).

### Prevalence

The prevalence of RA in AYAs worldwide increased from 34.11 per 100 000 population in 1990 to 36.34 per 100 000 population in 2019, with a statistically significant trend (AAPCs = 0.22, 95%CI: 0.19, 0.24, p<0.001). In terms of gender, in 1990, the prevalence of RA among males was 21.09 per 100 000 population, while it was 47.56 per 100 000 population among females. By 2019, the prevalence among males had increased to 22.97 per 100 000 population (AAPCs = 0.29, 95%CI: 0.26, 0.32, p<0.001), and among females, it had increased to 50.37 per 100 000 population (AAPCs = 0.19, 95%CI: 0.17, 0.22, p<0.001). Between 1990 and 2019, prevalence in the 10–14 year age group increased from 12.08 per 100 000 population to 12.41 per 100 000 population (AAPCs = 0.09, 95%CI: 0.06, 0.13, p< 0.001), in the 15–19 year age group from 32.17 per 100 000 population to 33.67 per 100 000 population (AAPCs = 0.14, 95%CI: 0.08, 0.2, p< 0·001), and in the 20–24 year age group from 60.14 per 100 000 population to 64.69 per 100 000 population (AAPCs = 0.25, 95%CI: 0.2, 0.31, p< 0.001).

When analyzing the influence of different SDI, it was found that the higher the SDI, the higher the prevalence. In high SDI areas, prevalence was 57.85 per 100 000 population (95%UI: 47.06, 70.86) in 1990 and 19.59 per 100 000 population (95%UI: 55.25, 82.01) in 2019, with an average annual increase in prevalence of 0.52% (95%CI: 0.55, 0.5, p<0.001), while in low SDI areas, prevalence was 19.59 per 100 000 population (95%UI: 13.89, 26.8) in 1990. In 2019, it was 20.92 per 100 000 population (95%UI: 14.89, 28.67), an average annual increase of 0.23% (95%CI: 0.25, 0.21, p<0.001). The region with the fastest increase in prevalence was the low-middle SDI region, which increased from 29.2 per 100 000 population in 1990 to 34.17 per 100 000 population in 2019 (AAPCs = 0.54, 95% CI: 0.48, 0.6, p<0.001).

At the regional level, Andean Latin America had the highest increase in prevalence, rising from 39.27 per 100 000 population in 1990 to 53.89 per 100 000 population in 2019 (AAPCs = 1.1, 95%CI: 1.09, 1.11, p<0.001), followed by Southern Latin America which increased from 34.07 per 100 000 population to 43.78 per 100 000 population (AAPCs = 0.87, 98%CI: 0.84, 0.9, p<0.001) (Table 2).

**Table 2:** The prevalence and AAPCs of rheumatoid arthritis among adolescents and young adults from 1990 to 2019.

	Prevalence per 100 000 population in 1990 (95% UI)	Prevalence per 100 000 population in 1990 (95% UI)	AAPCs (95% CI), 1990 to 2019	P value *
**Global**	34.11(25.95, 43.54)	36.34(27.49, 46.84)	0.22(0.19, 0.24)	< 0.001
**Sex**				
Male	21.09(15.72, 27.26)	22.97(17.08, 29.67)	0.29(0.26, 0.32)	< 0.001
Female	47.56(36.34, 60.57)	50.37(38.3, 65.17)	0.19(0.17, 0.22)	< 0.001
**Age group**				
10–14 years	12.08(8.25, 17.24)	12.41(8.44, 17.85)	0.09(0.06, 0.13)	< 0.001
15–19 years	32.17(24.13, 41.48)	33.67(25.16, 43.62)	0.14(0.08, 0.2)	< 0.001
20–24 years	60.14(46.04, 78.37)	64.69(49.17, 85.09)	0.25(0.2, 0.31)	< 0.001
**Sociodemographic index**				
High	57.87(47.06, 70.86)	67.25(55.25, 82.01)	0.52(0.5, 0.55)	< 0.001
High-middle	35.97(26.91, 46.2)	42(31.83, 53.93)	0.53(0.47, 0.58)	< 0.001
Middle	32.63(24.48, 42.46)	36.28(26.87, 47.61)	0.35(0.26, 0.44)	< 0.001
Low-middle	29.2(21.65, 38.35)	34.17(25.4, 44.92)	0.54(0.48, 0.6)	< 0.001
Low	19.59(13.89, 26.8)	20.92(14.89, 28.67)	0.23(0.21, 0.25)	< 0.001
**Region**				
Andean Latin America	39.27(25.52, 57.29)	53.89(34.75, 79.08)	1.1(1.09, 1.11)	< 0.001
Australasia	36.7(23.86, 53.08)	44.71(29.43, 64.69)	0.68(0.65, 0.72)	< 0.001
Caribbean	43.43(28.64, 63.29)	51.41(34.41, 74.63)	0.57(0.53, 0.61)	< 0.001
Central Asia	37.54(24.35, 55.7)	46.65(30.35, 69.43)	0.74(0.63, 0.85)	< 0.001
Central Europe	30.36(21.19, 42.04)	37.51(26.4, 51.88)	0.73(0.68, 0.79)	< 0.001
Central Latin America	50.1(37.14, 64.89)	64.28(48.03, 83.87)	0.85(0.81, 0.89)	< 0.001
Central Sub-Saharan Africa	16.53(9.88, 26.24)	17.4(10.57, 27.01)	0.16(0.1, 0.22)	< 0.001
East Asia	36.22(27.58, 45.7)	38.19(29.11, 47.98)	0.1(-0.21, 0.41)	0.52
Eastern Europe	25.97(19.03, 34.23)	29.42(21.75, 38.55)	0.4(0.25, 0.56)	< 0.001
Eastern Sub-Saharan Africa	15.1(10.02, 21.5)	16.14(10.71, 23.26)	0.22(0.19, 0.26)	< 0.001
High-income Asia Pacific	32.39(24.2, 41.3)	38.01(28.48, 48.74)	0.54(0.48, 0.6)	< 0.001
High-income North America	72.11(65.36, 79.52)	80.47(71.4, 91.06)	0.39(0.34, 0.44)	< 0.001
North Africa and Middle East	22.6(15.28, 31.86)	27.85(18.76, 39.52)	0.73(0.7, 0.77)	< 0.001
Oceania	23.6(15.03, 34.84)	25.43(16.38, 37.67)	0.26(0.24, 0.27)	< 0.001
South Asia	29.55(22.18, 38.17)	36.55(27.67, 47.18)	0.74(0.71, 0.77)	< 0.001
Southeast Asia	20.27(14.19, 27.81)	23.04(16.28, 31.26)	0.44(0.44, 0.45)	< 0.001
Southern Latin America	34.07(21.45, 50.3)	43.78(27.86, 63.9)	0.87(0.84, 0.9)	< 0.001
Southern Sub-Saharan Africa	30.45(22.23, 41.17)	31.26(22.74, 42.48)	0.05(-0.1, 0.21)	0.5
Tropical Latin America	68.63(54.36, 86.12)	85.84(68.27, 106.68)	0.78(0.75, 0.82)	< 0.001
Western Europe	65.91(47.01, 87.27)	72.2(51.67, 95.54)	0.31(0.3, 0.33)	< 0.001
Western Sub-Saharan Africa	8.86(5.65, 12.64)	10.08(6.6, 14.41)	0.45(0.41, 0.49)	< 0.001

UI = uncertainty interval. AAPCs = average annual percentage changes. CI = confidence interval.

* The P-value was determined by joinpoint regression analysis.

The joint point regression analysis of the changing trend of the prevalence found that the prevalence of RA among AYAs had great changes in 1997, 2000, 2009, 2014 and 2017, and the largest change occurred in 2014 (Fig 3B).

### DALYs rate

Globally, DALYs rate with RA were 5.96 per 100 000 population and 5.79 per 100 000 population for AYAs in 1990 and 2019, respectively, with an average decrease of 0.1 years per year (AAPCs = -0.1, 95%CI: -0.2, -0.01, p = 0.04).

In 1990, the DALYs rate of RA among males was 3.95 per 100 000 population, while it was 8.03 per 100 000 population among females. By 2019, the DALYs rate among males had decreased to 3.8 per 100 000 population (AAPCs = -0.14, 95%CI: -0.2, -0.09, p<0.001), and among females, it had decreased to 7.87 per 100 000 population (AAPCs = -0.07, 95%CI: -0.14, 0, p<0.001). Between 1990 and 2019, DALYs rate in the 10–14 year age group decreased from 2.71 per 100 000 population to 2.33 per 100 000 population (AAPCs = -0.54, 95%CI: -0.72, -0.37, p< 0.001), in the 15–19 year age group from 5.72 per 100 000 population to 5.42 per 100 000 population (AAPCs = -0.18, 95%CI: -0.26, -0.09, p< 0.001), and in the 20–24 year age group increased from 9.75 per 100 000 population to 9.85 per 100 000 population (AAPCs = 0.03, 95%CI: -0.04, 0.1, p = 0.4).

The analysis of DALYs in different SDI areas shows that the higher the SDI, the higher the DALYs rate. In high SDI areas, DALYs rate were 8.97 per 100 000 population (95%UI: 6.07, 12.7) in 1990 and DALYs rate were 9.91 per 100 000 population (95%UI: 6.61, 14.27) in 2019, with an average annual increase in DALYs rate of 0.35% (95%CI: 0.32, 0.37, p<0.001). In low SDI areas, DALYs rate were 3.46 per 100 000 population (95%UI: 2.29, 4.9) in 1990 and 3.48 per 100 000 population (95%UI: 2.25, 5.0) in 2019, representing an average annual increase of 0.01% (95%CI: -0.03, 0.06, p = 0.62).

DALYs for AYAs increased over the 20-year period in some regions, while they declined in others. The largest increase was in Southern Latin America, from 5.83 per 100 000 population in 1990 to 7.21 per 100 000 population in 2019 (AAPCs = 0.75, 95% CI: 0.68, 0.82, p< 0.001), followed by Tropical Latin America, increased from 11.22 per 100 000 people in 1990 to 13.54 per 100 000 people in 2019 (AAPCs = 0.65, 95%CI: 0.59, 0.71, p< 0.001). The largest decline was in Southern Sub-Saharan Africa, from 7 per 100 000 to 5.46 per 100 000 (AAPCs = -0.91, 95%CI: -1.09, -0.72, p<0.001). DALYs decreased for AYAs aged 10–14 and 15–19 over a 20-year period, with AAPCs of -0.54 (95% CI: -0.37, -0.72, p<0.001) and -0.18 (95% CI: -0.09, -0.26, p<0.001), respectively. There was no significant change in DALYs among adolescents aged 20–24 (AAPCs = 0.03, 95% CI: 0.1, -0.04, p = 0.40) (Table 3).

**Table 3:** The DALYS rate and AAPCs of rheumatoid arthritis among adolescents and young adults from 1990 to 2019.

	DALYs per 100 000 population in 1990 (95% UI)	DALYs per 100 000 population in 2019 (95% UI)	AAPCs (95% CI), 1990 to 2019	p value *
**Global**	5.96(4.14, 8.3)	5.79(3.89, 8.27)	-0.1(-0.2, -0.01)	0.04
**Sex**				
Male	3.95 (2.8, 5.52)	3.8(2.57, 5.42)	-0.14(-0.2, -0.09)	< 0.001
Female	8.03 (5.49, 11.19)	7.87(5.28, 11.31)	-0.07(-0.14, 0)	0.05
**Age group**				
10–14 years	2.71 (1.91, 3.94)	2.33(1.56, 3.46)	-0.54(-0.72, -0.37)	< 0.001
15–19 years	5.72 (3.94, 8.02)	5.42(3.62, 7.81)	-0.18(-0.26, -0.09)	< 0.001
20–24 years	9.75 (6.67, 13.6)	9.85(6.49, 14.04)	0.03(-0.04, 0.1)	0.40
**Sociodemographic index**				
High	8.97(6.07, 12.7)	9.91(6.61, 14.27)	0.35(0.32, 0.37)	< 0.001
High-middle	6.35(4.39, 8.82)	6.7(4.51, 9.63)	0.17(0.09, 0.25)	0.00
Middle	6.02(4.23, 8.29)	5.91(3.98, 8.47)	-0.07(-0.18, 0.04)	0.20
Low-middle	5.12(3.51, 7.21)	5.46(3.63, 7.85)	0.22(0.18, 0.27)	< 0.001
Low	3.46(2.29, 4.9)	3.48(2.25, 5.08)	0.01(-0.03, 0.06)	0.62
**Region**				
Andean Latin America	7.52(4.96, 11.02)	8.65(5.19, 13.03)	0.47(0.38, 0.57)	< 0.001
Australasia	5.53(3.21, 8.72)	6.6(3.81, 10.36)	0.61(0.52, 0.69)	< 0.001
Caribbean	7.42(4.72, 11.22)	8.23(5.02, 12.39)	0.35(0.28, 0.43)	< 0.001
Central Asia	8.09(5.27, 11.69)	8.35(5.25, 12.23)	0.16(-0.02, 0.34)	0.09
Central Europe	5.58(3.79, 7.94)	5.93(3.79, 8.75)	0.2(0.14, 0.26)	< 0.001
Central Latin America	9.68(6.9, 13.14)	10.66(7.21, 14.99)	0.33(0.3, 0.36)	< 0.001
Central Sub-Saharan Africa	3.72(2.35, 5.44)	3.42(2.02, 5.2)	-0.34(-0.44, -0.24)	< 0.001
East Asia	6.96(4.95, 9.53)	6.47(4.47, 9.1)	-0.28(-0.5, -0.06)	0.01
Eastern Europe	5.49(4.01, 7.29)	5.5(3.92, 7.54)	0(-0.15, 0.15)	0.95
Eastern Sub-Saharan Africa	3.08(2.07, 4.39)	2.83(1.79, 4.23)	-0.31(-0.39, -0.22)	< 0.001
High-income Asia Pacific	5.75(3.98, 8.03)	6(3.94, 8.54)	0.14(0.08, 0.2)	0.00
High-income North America	10.67(7.24, 14.63)	11.75(8.02, 16.09)	0.34(0.29, 0.39)	< 0.001
North Africa and Middle East	3.86(2.41, 5.68)	4.36(2.63, 6.68)	0.43(0.36, 0.49)	< 0.001
Oceania	3.95(2.35, 6.06)	4.41(2.67, 6.68)	0.37(0.28, 0.47)	< 0.001
South Asia	4.55(2.99, 6.56)	5.49(3.6, 7.96)	0.63(0.59, 0.68)	< 0.001
Southeast Asia	3.68(2.5, 5.31)	3.85(2.5, 5.58)	0.17(0.11, 0.22)	< 0.001
Southern Latin America	5.83(3.57, 9.05)	7.21(4.34, 11.21)	0.75(0.68, 0.82)	< 0.001
Southern Sub-Saharan Africa	7(5.07, 9.22)	5.46(3.6, 7.69)	-0.91(-1.09, -0.72)	< 0.001
Tropical Latin America	11.22(7.78, 15.58)	13.54(9.29, 18.82)	0.65(0.59, 0.71)	< 0.001
Western Europe	10.19(6.52, 15.13)	10.62(6.55, 16.2)	0.13(0.11, 0.16)	< 0.001
Western Sub-Saharan Africa	1.73(1.1, 2.53)	1.96(1.27, 2.86)	0.44(0.35, 0.54)	< 0.001

DALYs = disability-adjusted life-years. UI = uncertainty interval. AAPCs = average annual percentage changes. CI = confidence interval.

* The P-value was determined by joinpoint regression analysis.

Joint point regression analysis found substantial changes in RA DALYs in the AYAs population in 1996, 1999, 2009, 2014 and 2017, with the largest change in 2014 (Fig 3C).

### Effects of different SDI on Incidence rate

**Fig 4:**
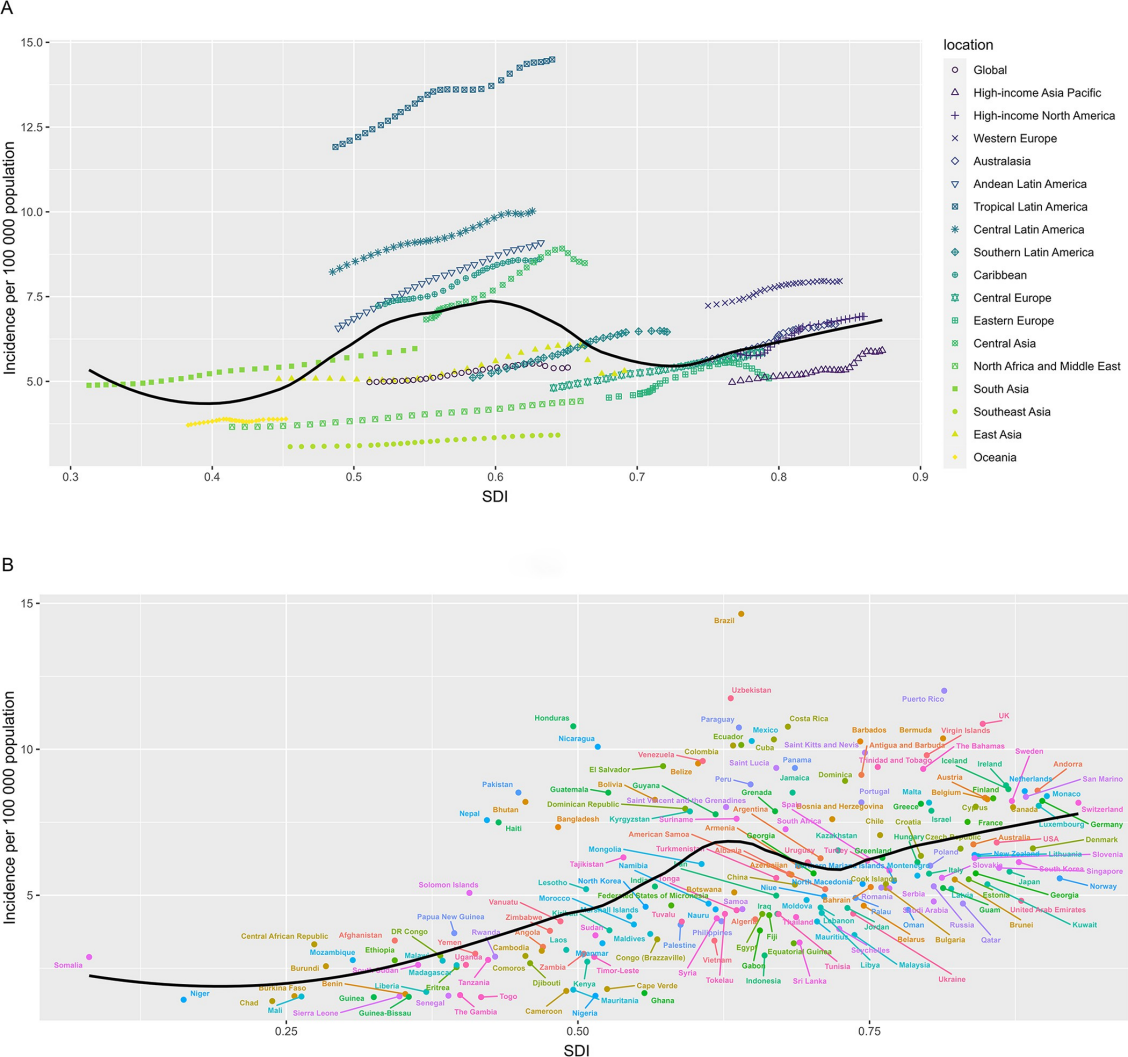
The association between the incidence of RA among AYAs and SDI at the regional level from 1990 to 2019 (A) and national level at 2019 (B). RA = rheumatoid arthritis. AYAs = adolescents and young adults. SDI = social development index.

At the regional level, there is a nonlinear relationship between the incidence rate and SDI. With the improvement of SDI, it rises and falls intermittently (Fig 4). During the period from 1990 to 2019, the super-regions of Western Europe, High-income North America, and High-income Asia Pacific showed an upward trend. All regions in the Latin American super-region showed an upward trend during the period from 1990 to 2019, and were higher than expected. During the period from 1990 to 2019, the incidence rates in East Asia, Eastern Europe, North Africa and the Middle East, Southeast Asia, Oceania, and High-income Asia Pacific were lower than expected (Fig 4A).

The analysis at the national level found that there is a nonlinear relationship between the incidence rate and SDI, and the high burden of RA not only exists in developed countries, but also in underdeveloped countries. The incidence rates in Puerto Rico, the UK, Uzbekistan, Brazil, Uzbekistan, Honduras, Nicaragua, Pakistan, Nepal, and many other countries are much higher than expected. In contrast, many countries, such as Norway, the United Arab Emirates, Qatar, Sri Lanka, Nigeria, Senegal, have lower than expected incidence rates (Fig 4B).

## Discussions

RA is a chronic inflammatory condition that not only affects the musculoskeletal system but also has negative impacts on multiple systems and organs in the body, as highlighted in several studies [1, 14–16]. Therefore, early intervention and treatment for RA in AYAs is of utmost importance. Several studies have investigated the global burden and trends of RA in adolescents and young adults [9, 17], but to our knowledge, this study is the first systematic evaluation of the global burden and trends of RA in adolescents and young adults across 204 countries and territories from 1990 to 2019.

The study revealed that in 2019, the global incidence of RA in adolescents was 5.41 per 100 000 population, and the prevalence was 36.34 per 100 000 population. Compared to 1990, these rates increased by 8.63% and 6.54%, respectively. Although there was a slight decrease of 2.94% in DALYs rate between 1990 and 2019, the change was not statistically significant. Overall, the trend of RA incidence and disease burden in the adolescent population remains concerning, necessitating more effective intervention measures to curb this phenomenon and reduce future disease burden.

Analysis of the incidence, prevalence, and DALYs rate of RA in different genders and age groups among adolescents revealed that females had higher incidence and prevalence as well as DALYs rate compared to males. This is consistent with the findings of studies conducted by Guan S. et al [2] and Safiri S. et al [9]. Similar gender disparities were also found in middle-aged and older populations. It is well-documented that autoimmune diseases are more common in females, and they are the fourth leading cause of disability in women [18, 19]. This may be related to sex hormones, as androgens have been shown to suppress humoral and cellular immune responses, while estrogens enhance humoral immune responses [20]. A study of 105 303 men with prostate cancer found that those who received androgen deprivation therapy had a 23 percent increase in RA diagnoses over five years [21]. Another study on RA patients found lower levels of androgens (testosterone, dihydrotestosterone, and dehydroepiandrosterone sulfate) in their body fluid, suggesting that reduced androgen levels increase the risk of RA diagnosis [22]. Therefore, in formulating policies for the prevention and treatment of RA, greater attention should be paid to the female population. Additionally, androgen intervention may become a useful treatment measure for RA.

When analyzing the relationship between SDI and incidence rates in different regions and countries, it was found that the relationship between the incidence of rheumatoid arthritis (RA) in the adolescent population and SDI exhibits a complex nonlinear association. The burden of RA is not limited to developed or underdeveloped countries, and there are reports of high RA burdens in various SDI countries. The differences in RA burden among different SDI regions are consistent with the findings of Guan SY et al. [2]. Safiri S et al. also discovered this complex nonlinear relationship when analyzing the global burden of rheumatoid arthritis through the GBD 2017 [9]. Even within the same super-region, there are differences in RA incidence rates among different regions or countries. In Southeast Asia, including Cambodia and Thailand, the incidence rates are relatively low, while in South Asia, such as India and Pakistan, the incidence rates are relatively high. Currently, there is no clear evidence to provide a reasonable explanation for this phenomenon. Potential trends in environmental risk factors and differences in medical policies among different countries may affect the incidence of RA [23]. Smoking, as one of the important risk factors, requires precise monitoring and specific prevention plans to be developed by each country. Whether other potential risk factors, such as obesity, intestinal microbiota, diet, and genetic factors, will affect the incidence of RA still requires further research [9, 24].

Although the overall trend of the incidence and prevalence of RA in adolescents and young adults has been increasing from 1990 to 2019, a significant inflection point occurred in 2014 when the trend shifted from an upward trajectory to a downward trend, representing the largest change in the 30-year period. The decline in DALYs rate was slight since 2009, with the largest decrease also observed in 2014. Overall, there has been little significant change in DALYs rate from 1990 to 2017. The magnitude of DALYs largely depends on the effectiveness of RA treatment and the importance of early intervention. Over the past few decades, the treatment of RA has evolved to emphasize the importance of early intervention, and newer and more effective drugs have been applied to patients, effectively limiting disease activity, radiographic progression, and functional loss [25–27]. A Norwegian study found a general decline in the incidence of joint replacement, joint fusion, and synovectomy among RA patients between 1997 and 2012. The general increasing trend in the use of synthetic and biological disease-modifying antirheumatic drugs (DMARDs) is consistent with less joint destruction and improved long-term outcomes in RA patients [28]. However, due to the higher costs involved, the benefits of this may be more evident in high-income regions. Early diagnosis and treatment can prevent the progression of joint damage in 90% of early RA patients [26]. In underdeveloped regions, the lack of standardized diagnosis makes it difficult to diagnose RA early. Delayed diagnosis of RA in these regions may hinder effective treatment and eventually lead to RA-related disabilities. Therefore, it is crucial to raise awareness of the burden of RA in underdeveloped regions and improve outcomes by implementing early clinical diagnosis and utilizing relatively low-cost drugs, such as methotrexate, to manage the disease, prevent RA-related complications, and reduce the burden and adverse consequences of RA [9].

The study has several limitations. Firstly, the data for this research relies on the GBD 2019 database. Therefore, the quality of this study is subject to limitations such as variations in the quality and availability of GBD 2019 data. The data collected from different regions and countries may differ in terms of quality, comparability, accuracy, and extent of missing data. Secondly, various countries, particularly underdeveloped ones, may face challenges in effectively screening, assessing, and treating RA. This can restrict the representativeness of the statistical data.

## Conclusion

This study found an overall increase in the incidence and prevalence of RA among AYAs over the past 30 years, while DALYs rate have remained virtually unchanged. In addition, the incidence, prevalence and DALYs rate of RA increased with the increase of age. Fortunately, recent proactive preventive measures and treatment methods have shown promising results. In the future, it is necessary to pay more attention to female populations and AYAs patients in order to further reduce the global burden of RA.

## Supporting information

S1 Table Raw data on the incidence, prevalence, and DALYs rates of rheumatoid arthritis in adolescents and young adults at the regional level from 1990 to 2019. (XLSX)

S2 Table Raw data on the incidence, prevalence, and DALYs rates of rheumatoid arthritis in adolescents and young adults at the national level from 1990 to 2019. (XLSX)

## Abbreviations

AAPCs: average annual percentage changes
APC: annual percent changes
AYAs: adolescents and young adults
CI: confidence intervals
DALYs: disability-adjusted life-years
GBD: Global Burden of Diseases
RA: rheumatoid arthritis
SDI: socio-demographic index
UI: Uncertainty intervals
